# Distinctive Clinical Effects of Haemorrhagic Markers in Cerebral Amyloid Angiopathy

**DOI:** 10.1038/s41598-017-16298-1

**Published:** 2017-11-22

**Authors:** Young Kyoung Jang, Hee Jin Kim, Jin San Lee, Yeo Jin Kim, Ko Woon Kim, Yeshin Kim, Hyemin Jang, Juyoun Lee, Jong Min Lee, Seung-Joo Kim, Kyung-Ho Yu, Andreas Charidimou, David J. Werring, Sung Tae Kim, Duk L. Na, Sang Won Seo

**Affiliations:** 1 Department of Neurology, Samsung Medical Center, Sungkyunkwan University School of Medicine, Gangnam-gu, Seoul 06351 Korea; 2 Neuroscience Center, Samsung Medical Center, Sungkyunkwan University School of Medicine, Gangnam-gu, Seoul 06351 Korea; 3 Department of Neurology, Kyung Hee University Hospital, Gangnam-gu, Seoul Korea; 4 Department of Neurology, Chuncheon Sacred Heart Hospital, Chuncheon, South Korea; 5 Department of Neurology, Chonbuk National University Hospital, Chun-Ju, South Korea; 6 Department of Neurology, Chungnam National University School of Medicine, Daejeon, Korea; 7 Department of Biomedical Engineering, Hanyang University, Korea; 8 Department of Neurology, Hallym University College of Medicine, Hallym Neurological Institute, Seoul, South Korea; 9 Hemorrhagic Stroke Research Program, Department of Neurology, Massachusetts General Hospital, Harvard Medical School, Boston, MA USA; 10 Institute of Neurology, University College London, London, UK; 11 Department of Radiology; Samsung Medical Center, Sungkyunkwan University School of Medicine, Gangnam-gu, Seoul 06351 Korea; 12 Department of Health Sciences and Technology, SAIHST, Sungkyunkwan University, Seoul, Republic of Korea; 13 Department of Clinical Research Design & Evaluation, SAIHST, Sungkyunkwan University, Seoul, Republic of Korea

## Abstract

Restricted lobar cerebral microbleeds (CMBs) and cortical superficial siderosis (CSS) are the characteristic markers of cerebral amyloid angiopathy (CAA). However, their effects on clinical features has not been evaluated well. The purpose of this study is to investigate the clinical implication of these markers in clinical-radiologically diagnosed CAA. A total of 372 patients with possible or probable CAA who met the modified Boston criteria were recruited in a memory clinic setting. Cortical thickness was measured using surface based methods. Presence of restricted multiple lobar CMBs were independently associated with cortical thinning across the entire cortical regions while presence of CSS was independently associated with cortical thinning primarily in the bilateral frontal region. Presence of restricted multiple lobar CMBs was associated with impairment in all cognitive domains such as attention, language, visuospatial, memory and frontal executive functions while presence of CSS was associated with attention and frontal dysfunction. The relationships of restricted multiple lobar CMBs or CSS with cognitive impairment were partially mediated by thinning in the corresponding cortical regions. Our findings suggested that restricted multiple lobar CMBs and CSS affect distinctive clinical features, providing new insights into potential mechanisms in CAA.

## Introduction

Cerebral microbleeds (CMBs) are defined as small, round dark-signal lesions on T2* gradient-echo (GRE) magnetic resonance imaging (MRI)^1^, and preferentially located in lobar area in CAA^2^. Cortical superficial siderosis (CSS), which is linear deposits of the blood-breakdown residues within the subarachnoid space, the leptomeninges and the superficial layers of cerebral hemisphere. Strictly lobar CMBs and CSS have been suggested as characteristic neuroimaging markers of cerebral amyloid angiopathy (CAA) and have been recently adopted as clinical diagnostic markers even without intracranial haemorrhage (ICH)^3^. In fact, 90% of symptomatic CAA patients diagnosed by the presence of multiple lobar CMBs without ICH harboured moderate to severe CAA on neuropathology^4^. In addition, CSS was commonly (60%) observed in pathologically confirmed CAA patients^3^.

Previous studies showed that 60% of pathologically diagnosed CAA patients have cognitive impairment^5^, and CAA pathology seems to be associated with cognitive impairment independently of Alzheimer’s disease (AD) or other neurodegenerative pathologies^6–8^. A recent study also suggested that CAA might show cortical atrophy^9^, which has an important role in cognitive impairment. Given that lobar CMBs and CSS may arise from distinct vasculopathic mechanisms (involvements in cortical vessels for lobar CMBs versus leptomeningeal vessels for CSS)^3,10^, lobar CMBs and CSS may affect distinctively clinical features. Furthermore, it would be reasonable to expect that multiple lobar CMBs and CSS might be associated with cortical thinning, which in turn leads to cognitive impairment because cortical atrophy may be final common pathway of amyloid beta (Aβ) and cerebrovascular disease for cognitive impairments^11^.

In this study, we investigated the relationship between CAA haemorrhagic markers and cognition in a unique cohort of clinical-radiological CAA patients. We hypothesized that CAA haemorrhagic markers distinctively affected clinical features including cortical thickness and cognitive impairments. We also hypothesized that cortical thinning mediated the relationships between CAA haemorrhagic markers and cognition.

## Results

### Demographic and clinical characteristics

**Figure 1:**
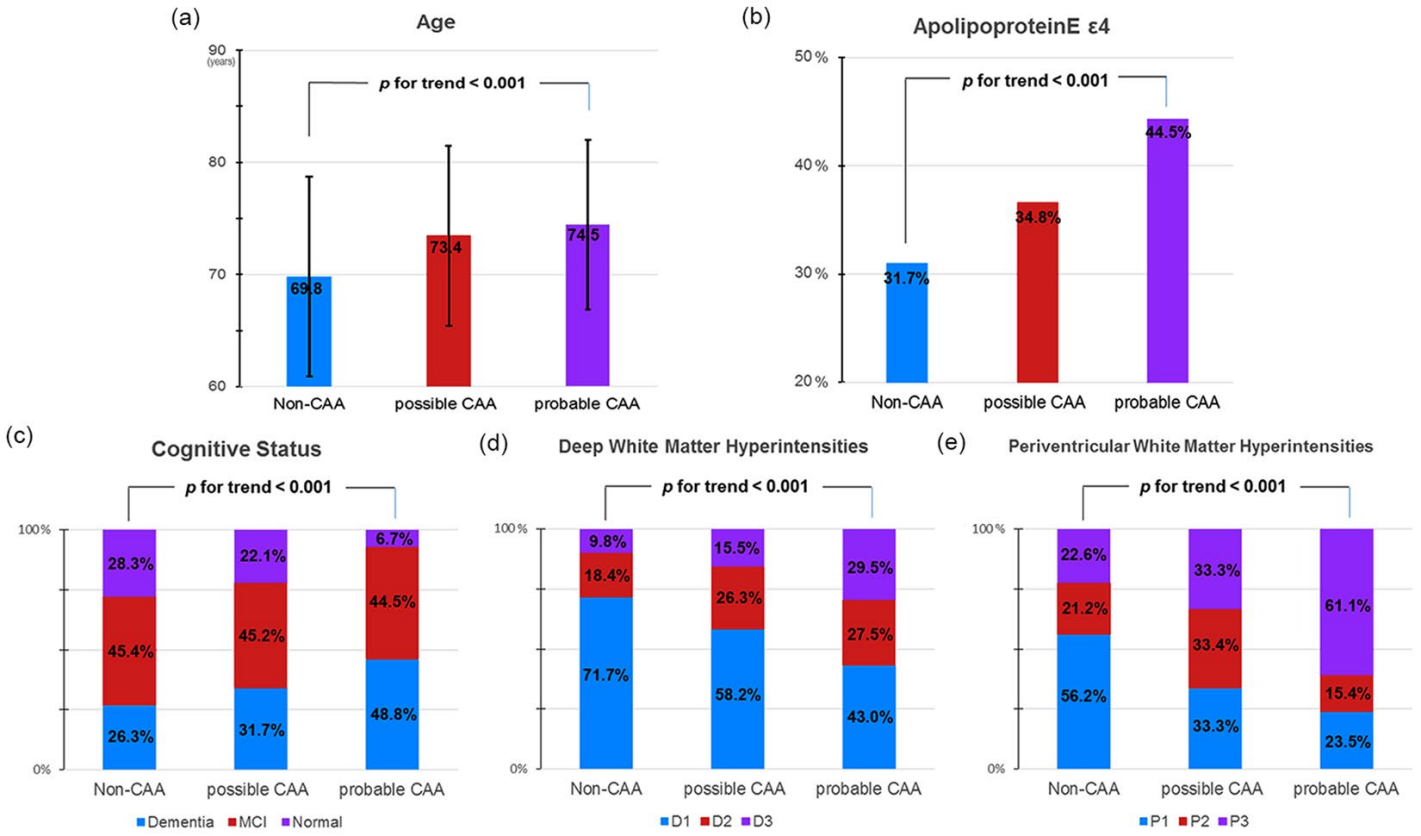
Trend analyses of clinical characteristics. Figure showed a significant trend of older age (**a**), a higher frequency of *APOE* ε4 (**b**), poorer cognitive status (**c**), and more severe deep (**d**) and periventricular (**e**) white matter hyperintensities among non-CAA, possible CAA, and probable CAA groups in order. CAA cerebral amyloid angiopathy.

Of 3,216 patients, 372 (11.6%) patients met the possible (n = 208, 6.5%) or probable (n = 164, 5.1%) CAA criteria^3^. Their demographic and clinical characteristics are summarized in Supplementary Table 1. Linear trend tests showed a significant trend for older age, a higher frequency of *APOE* ε4, poorer cognitive status, and more severe WMH among non-CAA, possible CAA, and probable CAA groups respectively (Fig. 1).

### Multiple lobar CMBs, CSS presence, and cortical thickness

There were no differences in demographics and vascular risk factors between CAA with multiple lobar CMBs (n = 150) and those without multiple lobar CMBs (n = 222) (Table 1). CAA with multiple lobar CMBs had a higher frequency of *APOE* ε4 allele than CAA without multiple lobar CMBs (*P* < 0.003). There were no differences in demographics and vascular risk factors between CAA with CSS (n = 73) and CAA without CSS (n = 299) (Table 1).

**Table 1:** Clinical characteristics, neuropsychological and imaging findings according to status of lobar CMBs and CSS in clinical-radiological CAA group.

	CAA with multiple lobar CMBs (n = 150)	CAA without multiple lobar CMBs (n = 222)	*P*	CAA with CSS (n = 73)	CAA without CSS (n = 299)	*P*
Age (years)	74.3 ± 7.6	73.7 ± 8.0	0.538	74.3 ± 6.3	73.8 ± 8.2	0.626
Education (years)	10.1 ± 5.7	9.9 ± 5.5	0.800	10.1 ± 5.7	10.0 ± 5.6	0.858
Sex (male) (n (%))	88 (39.1%)	61 (41.5%)	0.646	35 (47.9%)	114 (38.1%)	0.125
Risk factors						
Hypertension (n (%))	48 (32.7%)	76 (33.8%)	0.822	29 (39.7%)	95 (31.8%)	0.196
Diabetes (n (%))	76 (51.7%)	103 (45.8%)	0.264	29 (39.7%)	150 (50.2%)	0.109
Hyperlipidemia (n (%))	35 (23.8%)	64 (28.4%)	0.323	13 (17.8%)	86 (28.8%)	0.058
Cardiac disease (n (%))	22 (15.0%)	38 (16.9%)	0.622	13 (17.8%)	47 (15.7%)	0.663
Stroke (n (%))	20 (13.6%)	16 (7.1%)	0.098	6 (8.1%)	30 (10.0%)	0.638
Apolipoprotein E						
*APOE* ε4 (n (%))	64/142 (45.1%)	77/220 (35.0%)	0.055	28/70 (40.0%)	113/292 (38.7%)	0.841
*APOE* ε2 (n (%))	12/142 (8.5%)	27/220 (12.3%)	0.299	10/70 (13.7%)	29/292 (9.9%)	0.291
Presence of CSS (n (%))	37 (24.7%)	36 (16.2%)	0.044			
Presence of multiple lobar CMBs (n (%))				37 (50.6%)	113 (37.8%)	0.044

CMBs cerebral microbleeds; CSS cortical superficial siderosis; CAA cerebral amyloid angiopathy; n number; *APOE* Apolipoprotein E; *P P-*value.

**Figure 2:**
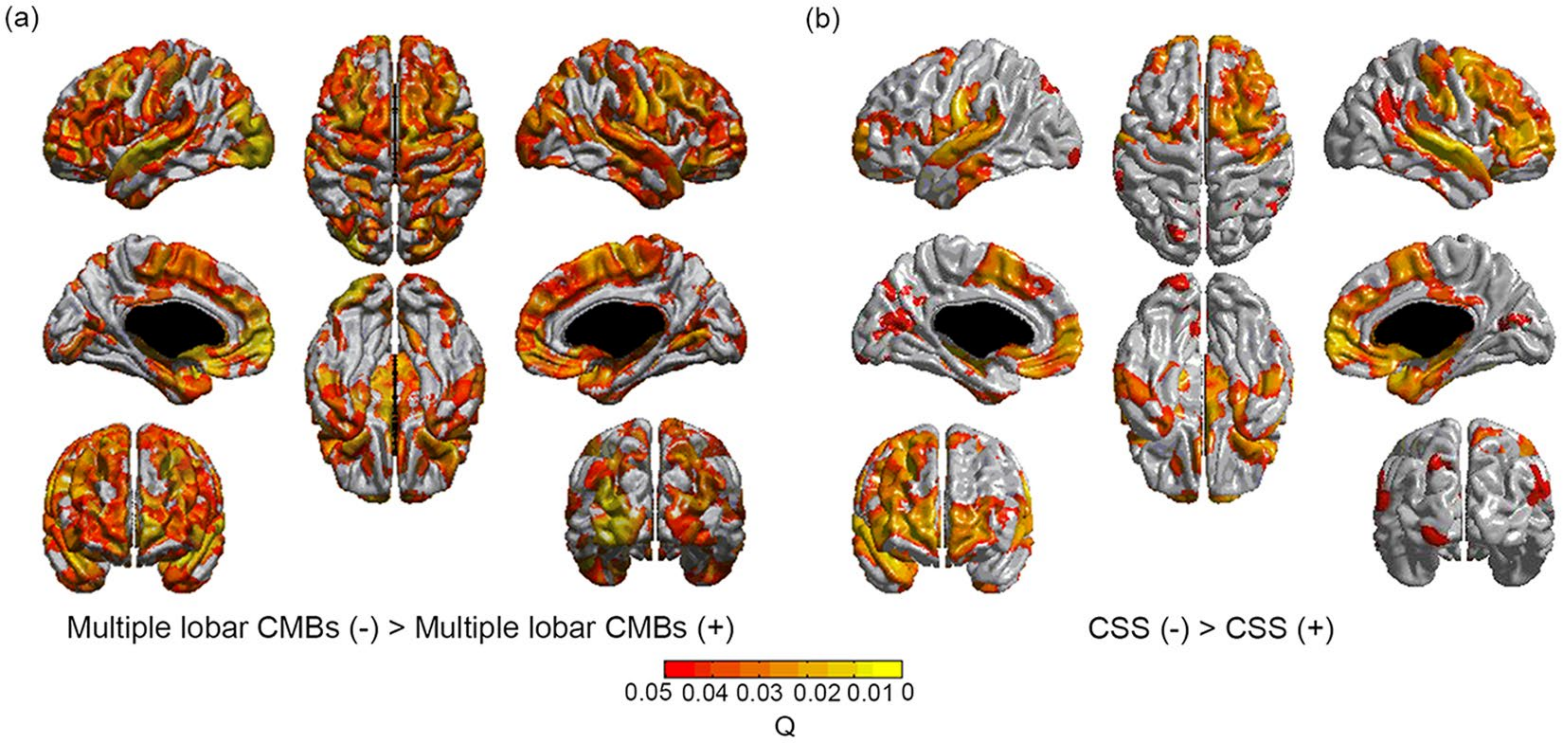
Topography of cortical thinning. Multiple lobar CMBs were associated with cortical thinning in bilateral medial and lateral frontal, medial and lateral temporal, lateral parietal, and occipital regions. (**a**) CSS was associated with cortical thinning in bilateral medial and lateral frontal, medial temporal and occipital and right lateral temporal regions. (**b**) Multiple lobar CMBs, and CSS were entered in a general linear model. Age, sex, education, intracranial volume, white matter hyperintensities, and another haemorrhagic marker were entered as covariates. CMBs cerebral microbleeds; CSS cortical superficial siderosis.

CAA with multiple lobar CMBs showed cortical thinning in the bilateral medial and lateral frontal, medial and lateral temporal, lateral parietal, and occipital regions compared to CAA without multiple lobar CMBs. CAA with CSS showed cortical thinning in the bilateral medial and lateral frontal, medial temporal and occipital and right lateral temporal and lateral parietal regions, compared to the CAA without CSS (Fig. 2b). Compared to CAA without multiple lobar CMBs, CAA with multiple lobar CMBs had decreased mean thickness in all cortical regions while compared to CAA without CSS, CAA with CSS also demonstrated decreased mean thickness in the frontal and temporal (Table 2).

**Table 2:** Effects of CAA haemorrhagic markers on cortical thickness and cognitive function in clinical-radiological CAA group.

	Multiple lobar CMBs			CSS		
	β	SE	*P*	β	SE	*P*
Cortical thickness*						
Frontal	−0.054	0.020	0.008	−0.054	0.026	0.037
Temporal	−0.051	0.025	0.045	−0.070	0.032	0.020
Parietal	−0.046	0.021	0.027	−0.043	0.026	0.109
Occipital	−0.037	0.018	0.044	−0.036	0.023	0.129
Cognitive function**						
Attention	−0.247	0.219	0.047	−1.093	0.269	<0.001
Language	−2.514	1.226	0.041	−2.260	1.521	0.138
Visuospatial function	−2.660	0.945	0.005	−1.028	1.158	0.375
Memory	−4.901	2.216	0.028	−3.138	2.762	0.257
Frontal executive function	−13.405	4.848	0.006	−12.071	5.874	0.032

CAA cerebral amyloid angiopathy; CMBs cerebral microbleeds; CSS cortical superficial siderosis; β unstandardized beta coefficient; SE standard error; *P P*-value; *Model adjusted for Age, Sex, Education, intracerebral volume, white matter hyperintensities, and CSS (+) (for lobar CMBs analyses) or multiple lobar CMBs (+) (for CSS (+) analyses) as covariates. **Model adjusted for Age, sex, education, white matter hyperintensities, and CSS (+) (for lobar CMBs analyses) or lobar CMBs (+) (for CSS (+) analyses) as covariates.

### Multiple lobar CMBs, CSS presence, and cognitive impairment

CAA with multiple lobar CMBs had worse performances in all cognitive domains than CAA without multiple lobar CMBs. Compared to the CAA without CSS, CAA with CSS also showed more impairment in the attention, frontal executive function and MMSE (Table 2).

### Effects of lobar CMBs and CSS on cognitive impairments through cortical thickness

**Figure 3:**
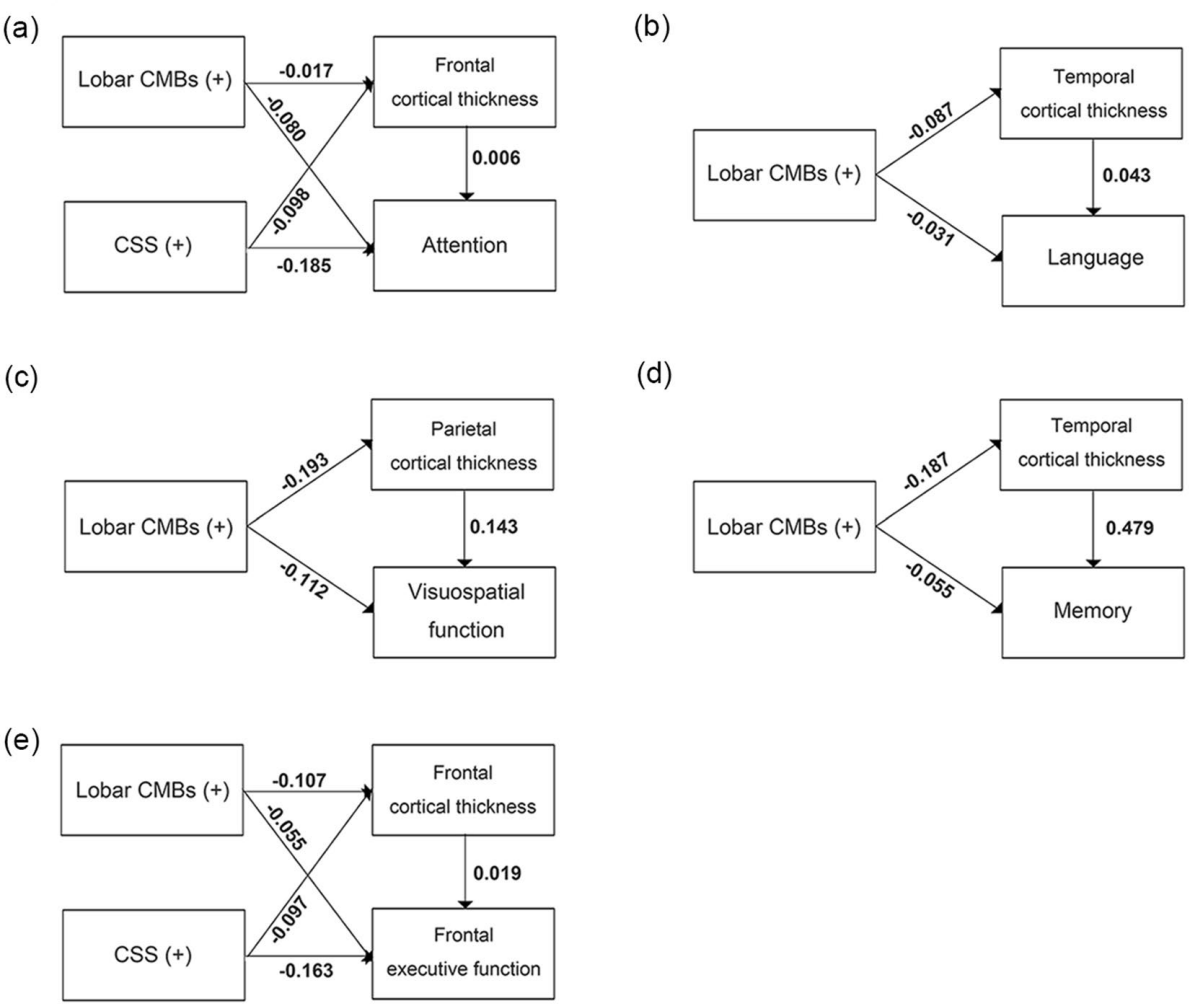
Path analyses for cognition. Multiple lobar CMBs and/or CSS were associated with attention (**a**), language (**b**), visuospatial (**c**), memory (**d**), and frontal executive (**e**) dysfunctions partially mediated by cortical thickness of corresponding area, respectively. The numbers on the paths are standardized coefficients which showed statistical significances. Cortical thickness was entered as a mediator variable for cognitive function. Multiple lobar CMBs, and CSS were entered as predictors. Age, sex, education, and white matter hyperintensities entered as covariates. The numbers on the paths are standardized coefficients. Direct paths that were statistically significant were reported. CMBs cerebral microbleeds; CSS cortical superficial siderosis.

Path analyses with adjustment for age, sex, education, and WMH showed that multiple lobar CMBs were associated with all cognitive domains partially mediated by cortical thickness of corresponding area, respectively (Fig. 3a–e). Path analyses showed that CSS presence was associated with decreased scores in attention, and frontal executive function partially mediated by frontal and total cortical thickness after adjustment for age, sex, education, and WMH (Fig. 3a,e) (Supplementary Table 2).

## Discussion

In this study, we investigated the clinical significance of CAA haemorrhagic markers in a large sample of patients fulfilling clinical-radiological criteria for CAA in a memory clinic setting. We found that multiple lobar CMBs were associated with impairment in multiple cognitive domains while CSS was correlated primarily with frontal dysfunction. Furthermore, the relationships of multiple lobar CMBs or CSS with cognitive impairment was partially mediated by thinning in the corresponding cortical regions. Taken together, our findings suggest that multiple lobar CMBs and CSS affect distinctive clinical features, providing new insights into potential mechanisms in CAA.

Among 3,216 memory clinic patients, 208 (6.5%)/164 (5.1%) of elderly people over 55 years of age fulfilled the Boston criteria for possible/probable CAA in our cohort. Lobar CMBs were observed in 10.6% and 44.0% of them had multiple lobar CMBs. Also, 2.7% of patients showed CSS. Our findings were generally consistent with previous studies showing that 13.7% of general population had lobar CMBs^12^ and 3% of memory clinic patients had CSS^13^.

We found that older age, a higher frequency of *APOE* ε4, poorer cognitive status, and more severe deep and periventricular WMH were the most prominent in probable CAA by possible CAA and non-CAA, which are consistent with previous studies. Furthermore, their differences between possible CAA and non-CAA groups occurred. Considering almost of all our possible CAA patients have only a single lobar CMB, our findings might potentiate the concept that a single lobar CMBs at least observed in memory clinic patients may represent an early sign of a widespread vasculopathy rather than just misidentification of normal vessel structures^4^.

Our first major finding was that the association between multiple lobar CMBs or CSS and cognitive impairment was distinct in terms of the specific cognitive domains affected. Multiple lobar CMBs were associated with cognitive impairment in multiple domains while CSS was correlated primarily with frontal dysfunction. Previous studies have shown inconsistent results of the clinical influence of CMBs^14,15^. A recent study from our group showed that changes in lobar, but not deep, CMBs were associated with cognitive decline over three years^16^. Studies based on CAA patients, however, consistently reported that CAA patients showed cognitive impairment^6–8^. Given that multiple lobar CMBs reflect a severe underlying CAA, multiple lobar CMBs might be associated with decreased cognition. In fact, several studies suggested that lobar CMBs were associated with frontal executive function^17^, or impairment in multiple cognitive domains^18^. Although CSS is known to be related to impaired cognition^19^, the specific pattern of cognitive impaired has not been investigated to date.

We also found that multiple lobar CMBs were associated with thinning in entire cortical regions, while CSS were associated with thinning in the frontal and temporal regions. To our knowledge, the relationship between focal haemorrhagic imaging markers and more diffuse brain structural changes has not been established yet. A recent study revealed that CAA patients had structural network disruption and neurological dysfunction^6^. A previous study also showed that lobar CMBs affected gray matter atrophy and hypometabolism in the temporal lobe^20^. In contrast to the present study, a recent study revealed that decreased cortical thickness in CAA patients did not correlate with lobar CMB, but related to impaired vascular reactivity^9^. This discrepancy most likely reflects differences in study population (primarily MCI or dementia patients in our sample compared with non-demented ICH subjects in that study, with high numbers of lobar CMBs). However, in the present study, associations between haemorrhagic markers and cortical thinning remained significant after controlling for WMH that might be possible confounder. Our findings, therefore, suggested that these CAA haemorrhagic markers are considered not only a radiologic indicator of the presence of underlying CAA but also determinant of the clinical phenotypes of CAA.

Our second major finding was that cortical thinning partially mediated the associations between key haemorrhagic imaging markers of CAA and cognitive impairment in the corresponding domains. The exact mechanisms by which these CAA haemorrhagic markers lead to cognitive impairment remain to be elucidated. However, given that haemorrhagic imaging marker burden might reflect more severe underlying CAA pathology^4^, they are likely to be associated with more parenchymal Aβ or cortical micro-infarcts. Parenchymal Aβ burden or micro-infarcts are also reported to be associated with cortical thinning as well as cognitive impairment^21,22^. Furthermore, previous studies from our group have shown that parenchymal Aβ burden affected cognitive impairment with and without the mediation of cortical thinning^11^. Therefore, our findings suggested cortical thinning might play an important role in the development of cognitive impairments in patients with CAA.

The strengths of our study are its standardized MRI imaging and neuropsychological protocols in a large sized CAA cohort in a memory clinic. However, some limitations need to be acknowledged. The main limitations of our study include the lack of pathological data of our clinical-radiologically diagnosed CAA patients. We also note that the sensitivity and specificity of a possible CAA diagnosis, based on the presence of a single lobar CMBs might be low. Considering that CAA with haemorrhagic markers reflect moderate to severe form of CAA, our data should be interpreted with caution. Finally, we did not measure micro-infarcts and determine whether micro-infarcts affect our findings.

Despite these limitations, our findings suggest that CAA might be classified into several phenotypes based on advanced neuroimaging and according to the presence and burden of key haemorrhagic markers of the disease. Our findings are hypothesis-generating and after further validation in other cohorts and clinical settings, will advance our understanding of potential CAA subtypes with relevance for designing and interpreting future treatment trials.

## Methods

### Study population

A total of 3,216 participants were enrolled in the Samsung Medical Center Neuroimaging Study Registry from July 2007 to December 2013. Clinical diagnosis was established at a multi-disciplinary conference applying standard research criteria for SMI, MCI and dementia syndromes^23–25^. Our recruitment was based on memory clinic enriching for patients with clinical Alzheimer’s disease, subcortical vascular dementia, MCI, or SMI and only a small minority had frontotemporal lobar dementia, dementia with Lewy body, or other degenerative dementia. This study employed a standardized diagnostic assessment protocol including structural magnetic MRI for neurodegeneration and cerebrovascular disease and detailed neuropsychological tests.

Using this registry, we identified 372 patients with possible or probable CAA who met the modified Boston criteria, based on the presence of CSS and/or lobar CMBs^3^.

We excluded patients with the presence of secondary causes of cognitive deficits (e.g., vitamin B_12_/folate, syphilis serology, and/or thyroid function issues), or the presence of structural lesions (e.g., territorial cerebral infarctions and brain tumours) on conventional brain MRI scans, or with psychiatric illnesses such as schizophrenia.

### Standard protocol approvals, registrations, and patient consent

We obtained written informed consent from each patient. This study was approved by the Institutional Review Board at the Samsung Medical Center. In addition, all methods were carried out in accordance with the approved guidelines.

### MRI acquisition

All participants underwent brain MRI including T2* GRE and three-dimensional (3D) T1 images at Samsung Medical Center using the same kind of 3.0 T MRI scanner (Philips 3.0 T Achieva; Best, the Netherlands). The following parameters were used for the T2* GRE images: axial slice thickness, 5.0 mm; inter-slice thickness, 2 mm; repetition time (TR), 669 ms; echo time (TE) 16 ms; flip angle, 18°; matrix size, 560 × 560 pixels. We acquired 3D T1 images with the following imaging parameters: sagittal slice thickness, 1.0 mm, over contiguous slices with 50% overlap; TR of 9.9 ms; TE of 4.6 ms; flip angle of 8°; and matrix size of 240 × 240 pixels, reconstructed to 480 × 480 over a field of view of 240 mm.

### Assessment of lobar CMBs and CSS

Lobar CMBs were defined as microbleeds located in lobar areas and meeting the following consensus criteria^1^: (1) homogeneous signal losses with blooming effects on T2* images, (2) round or ovoid lesions with a 10 mm diameter cut-off, (3) lesions at least half surrounded by brain parenchyma, and (4) distinct from other potential mimics such as iron or calcium deposits, or vessel flow voids.

CSS was defined as chronic blood residual products in superficial layers of the cerebral cortex or subarachnoid area^26^ with recommendation criteria as follows^27^: (1) bilinear track-like hypointensities on T2* images surrounding or within a cerebral sulcus; (2) absence of corresponding hyperintense signals on FLAIR images; (3) supratentorial location; and (4) exclusion of a previous ICH associated CSS (i.e., a CSS right beside an ICH).

Four experienced neurologists, who were blinded to clinical information rated lobar CMBs and CSS on T2* images. Intra-class correlation coefficient ranged from 0.87 to 0.91 for lobar CMBs and from 0.82 to 0.96 for CSS.

To estimate the severity of white matter hyperintensities (WMH) in deep and periventricular white matter, we used the Fazekas rating scale^28^.

### Cortical thickness data analyses

T1-weighted images were processed using the standard Montreal Neurological Institute anatomical pipeline. With a linear transformation, native MRI images were registered into a standardized stereotaxic space^29^. The N3 algorithm was used to correct the images for intensity-based non-uniformities^30^ caused by the non-homogeneities in the magnetic field. Then, the registered and corrected images were classified into white matter, gray matter, cerebrospinal fluid, and background, using a 3D stereotaxic brain mask and the Intensity-Normalized Stereotaxic Environment for Classification of Tissues algorithm. The surfaces of the inner and outer cortex were automatically extracted using the Constrained Laplacian-Based Automated Segmentation with Proximities algorithm^31^.

Cortical thickness values were calculated in the native space rather than Talairach space because of the limitations in linear stereotaxic normalization. As we transformed MR volumes in native space into stereotaxic space with a linear transformation matrix, the inverse transformation matrix was applied to the cortical thickness models to reconstruct them in native space^32^. Cortical thickness was defined as the Euclidean distance between the linked vertices of the inner and outer surfaces^31^. The thickness value was spatially normalized using surface-based two-dimensional registration with a sphere-to-sphere warping algorithm. Thus, the vertices of each subject were nonlinearly registered to a standard surface template^33,34^. Cortical thickness was subsequently smoothed using a surface-based diffusion kernel in order to increase the signal-to-noise ratio. We chose 20 mm full-width at half-maximum as the kernel size to maximize statistical power while minimizing false positives^35^. For global and lobar regional analysis, the data of 30 normal subjects that had previously been manually categorized to lobes with high inter-rater reliability^36^ were registered to the template. The template then took the label of maximum probability in each vertex.

The presence of extensive WMH in the MRI scans made it difficult to completely delineate the inner cortical surface with the correct topology due to tissue classification errors. To overcome this technical limitation, we automatically defined the WMH region using a FLAIR image and substituted it for the intensity of peripheral, normal-appearing tissue on the high-resolution T1 image after affine co-registration, as described in earlier studies^37^. Individual cortical surface of each subject was registered to the precategorized template and divided into frontal, temporal, parietal, and occipital lobes via automated processes. Averaged values of the thickness of the whole vertex in each hemisphere and lobar region were used for global analysis.

### Neuropsychological tests

All patients underwent neuropsychological tests using the Seoul Neuropsychological Screening Battery (SNSB)^38,39^. We derived domain composite scores using the summation of selective tests scores for each subdomain. The raw scores of digit span forward and backward made up the score of attention domain (total score: 17). The language domain score was derived from the raw score of the Boston Naming Test (total score: 60). The RCFT copy score were used for visuospatial function domain (total score: 36). The memory domain was calculated from the sum of immediate-recall score, delayed-recall score, and a recognition score from the Seoul Verbal Leaning and RCFT (total score: 168). Frontal executive function domain was conducted using phonemic and semantic controlled oral word association test (total score: unlimited).

### Statistical analysis

Chi-squared tests for categorical variables and t tests for continuous variables were performed to compare the CAA and non-CAA groups. Fisher exact test and global x^2^ for categorical variables and the analysis of variance for continuous variables were performed to compare the probable CAA, possible CAA, and non-CAA groups. Trend analyses of age, *APOE* genotype, cognition level and WMH severity were performed to evaluate tendency among the non-CAA, possible CAA and probable CAA groups. Both lobar CMBs and CSS were categorized according to a median number as dummy variables to classify this cohort into the severe and mild CAA groups; no or single lobar CMBs group versus multiple lobar CMBs group and CSS absent group versus CSS present group. To assess the effects of multiple lobar CMBs or CSS presence on cortical thickness, linear regression analyses were performed after controlling for age, sex, education, ICV, WMH and multiple lobar CMBs or CSS presence. To identify the effects of multiple lobar CMBs or CSS presence on cognition, we also used linear regression analyses after controlling for age, sex, education, WMH and lobar CMBs or CSS.

To investigate the topography of cortical thinning associated with multiple lobar CMBs or CSS presence, we entered these two markers simultaneously in a general linear model as predictors for regional cortical thickness on a vertex-by-vertex basis. Covariates included age, gender, education, ICV, WMH and another CAA haemorrhagic marker for each lobar CMBs or CSS. The resulting statistical maps were thresholded, using the false discovery rate theory at a Q value of 0.05, after pooling the probability values (*P*) from the regression analyses. Regional cortical thickness was compared using Matlab 7.11 for Windows (MathWorks, Natrick, MA, USA).

Path analyses were performed to investigate the relationships between CAA imaging markers, cortical thickness and cognitive impairment in corresponding each domain using AMOS after controlling for age, sex, education, and WMH.

All statistical analyses were performed with SPSS version 22.0 and, AMOS 18 (SPSS, Chicago, IL). Statistical significance was defined as two-tailed *P* < 0.05.

## Electronic supplementary material

Table 1
