# Author Correction: Distinctive Clinical Effects of Haemorrhagic Markers in Cerebral Amyloid Angiopathy

**DOI:** 10.1038/s41598-018-23383-6

**Published:** 2018-03-21

**Authors:** Young Kyoung Jang, Hee Jin Kim, Jin San Lee, Yeo Jin Kim, Ko Woon Kim, Yeshin Kim, Hyemin Jang, Juyoun Lee, Jong Min Lee, Seung-Joo Kim, Kyung-Ho Yu, Andreas Charidimou, David J. Werring, Sung Tae Kim, Duk L. Na, Sang Won Seo

**Affiliations:** 1 Department of Neurology, Samsung Medical Center, Sungkyunkwan University School of Medicine, Gangnam-gu, Seoul, 06351 Korea; 2 Neuroscience Center, Samsung Medical Center, Sungkyunkwan University School of Medicine, Gangnam-gu, Seoul, 06351 Korea; 3 Department of Neurology, Kyung Hee University Hospital, Gangnam-gu, Seoul, Korea; 4 Department of Neurology, Chuncheon Sacred Heart Hospital, Chuncheon, South Korea; 5 Department of Neurology, Chonbuk National University Hospital, Chun-Ju, South Korea; 6 Department of Neurology, Chungnam National University School of Medicine, Daejeon, Korea; 7 Department of Biomedical Engineering, Hanyang University, Seoul, Korea; 8 Department of Neurology, Hallym University College of Medicine, Hallym Neurological Institute, Seoul, South Korea; 9 Hemorrhagic Stroke Research Program, Department of Neurology, Massachusetts General Hospital, Harvard Medical School, Boston, MA USA; 10 Institute of Neurology, University College London, London, UK; 11 Department of Radiology, Samsung Medical Center, Sungkyunkwan University School of Medicine, Gangnam-gu, Seoul, 06351 Korea; 12 Department of Health Sciences and Technology, SAIHST, Sungkyunkwan University, Seoul, Republic of Korea; 13 Department of Clinical Research Design & Evaluation, SAIHST, Sungkyunkwan University, Seoul, Republic of Korea

Correction to: *Scientific Reports* 10.1038/s41598-017-16298-1, published online 22 November 2017

In the original version of this Article, Jong Min Lee was incorrectly affiliated with ‘Department of Biomedical Engineering, Nanyang University, Nanyang Dr, Singapore.’

The correct affiliation is listed below.

“Department of Biomedical Engineering, Hanyang University, Seoul, Korea”

This error has now been corrected in the PDF and HTML versions of the Article, and in the accompanying ‘Table 1’ file.

