# Supplementary material for: Distinctive Clinical Effects of Haemorrhagic Markers in Cerebral Amyloid Angiopathy
Source: Sci Rep. 2017 Nov 22;7:15984. doi: 10.1038/s41598-017-16298-1 (PMC5700189; doi:10.1038/s41598-017-16298-1)
Supplement: Supplementary file 1 — Table 1 [file 41598_2017_16298_MOESM1_ESM.doc]

**Distinctive Clinical Effects of Haemorrhagic Markers in Cerebral Amyloid Angiopathy**

Young Kyoung Jang,^1,2^ Hee Jin Kim,^1,2^ Jin San Lee,^3^ Yeo Jin Kim,^4^ Ko Woon Kim,^5^ Yeshin Kim,^1,2^ Hyemin Jang,^1,2^ Juyoun Lee,^6^ Jong Min Lee,^7^ Seung-Joo Kim,^1,2^ Kyung-Ho Yu,^8^ Andreas Charidimou,^9^ David J. Werring,^10^ Sung Tae Kim,^10^ Duk L. Na,^1,2,12^ Sang Won Seo*^1,2,12,13^

^1^Department of Neurology, ^11^Department of Radiology; Samsung Medical Center, Sungkyunkwan University School of Medicine; ^2^Neuroscience Center, Samsung Medical Center; ^3^Department of Neurology, Kyung Hee University Hospital; ^4^Department of Neurology, Chuncheon Sacred Heart Hospital; ^5^Department of Neurology, Chonbuk National University Hospital; ^6^Department of Neurology, Chungnam National University School of Medicine; ^7^Department of Biomedical Engineering, Hanyang University; ^8^Department of Neurology, Hallym University College of Medicine, Hallym Neurological Institute; ^9^Hemorrhagic Stroke Research Program, Department of Neurology, Massachusetts General Hospital, Harvard Medical School; ^10^Institute of Neurology, University College London; ^12^Department of Health Sciences and Technology, SAIHST, Sungkyunkwan University; ^13^Department of Clinical Research Design & Evaluation, SAIHST, Sungkyunkwan University

**Supplementary table 1**

Demographic and clinical characteristics of subjects

|  | **CAA** | | | **Non-CAA**  **(n = 2,844)** |
| --- | --- | --- | --- | --- |
|  | **Total CAA**  **(n = 372)** | **Probable CAA**  **(n = 164)** | **Possible CAA**  **(n = 208)** |
| **Age** (years) | 73.9 ± 7.9* | 74.5 ± 7.5* | 73.4 ± 8.1* | 69.8 ± 8.9 |
| **Education** (years) | 10.0 ± 5.6 | 10.1 ± 5.7 | 9.9 ± 5.5 | 10.0 ± 5.4 |
| **Sex (male)** (n (%)) | 149 (40.1%) | 69 (42.1%) | 80 (38.5%) | 987 (34.7%) |
| **Risk factors** |  |  |  |  |
| **Hypertension** (n (%)) | 124 (33.3%) | 58 (35.4%) | 66 (31.7%) | 819 (28.8%) |
| **Diabetes** (n (%)) | 179 (48.1%) | 83 (50.6%) | 96 (46.2%) | 1254 (44.1%) |
| **Hyperlipidemia** (n (%)) | 99 (26.6%) | 40 (24.4%) | 59 (28.4%) | 865 (30.4%) |
| **Cardiac disease** (n (%)) | 60 (16.1%) | 25 (15.2%) | 35 (16.8%) | 399 (14.0%) |
| **Stroke** (n (%)) | 36 (9.7%)^a^ | 21 (12.8%)^a^ | 15 (7.2%) | 181 (6.4%) |
| **Apolipoprotein E** |  |  |  |  |
| **Apolipoprotein E ε4** (n (%)) | 141/362 (39.0%)^a^ | 69/155 (44.5%)^a^ | 72/207 (34.8%)^b^ | 875/2829 (30.9%) |
| **Apolipoprotein E ε2** (n (%)) | 39/36 (10.8%) | 16/155 (10.3%) | 23/207 (11.1%) | 282/2829 (10.0%) |
| **Cognition level** (n (%)) | 146/167/59^a^ | 80/73/11^a^ | 66/94/48^a,b^ | 721/1247/77 |
| **(Dementia/MCI/Normal)** | (39.2%/44.9%/15.9%) | (48.8%/44.5%/6.7%) | (31.7%/45.2%/22.1%) | (26.3%/45.4%28.3%) |

**CAA** cerebral amyloid angiopathy; **n** number; **MCI** Mild cognitive impairment

^a^*P-*value<0.05 between the Non-CAA group and other groups; ^b^*P-*value<0.05 between the possible CAA and probable CAA group

**Supplementary table 2. Effects of lobar CMBs and CSS on cognitive impairments** through cortical thickness

|  | Standardized beta  (Unstandardized beta) | SE | *P* | Standardized beta  (Unstandardized beta) | SE | *P* |
| --- | --- | --- | --- | --- | --- | --- |
|  | **Frontal cortical thickness** | | | **Attention** | | |
| **Multiple lobar CMBs** | -0.163 (-0.073) | 0.021 | <0.001 | -0.050 (-0.163) | 0.224 | 0.004 |
| **CSS** | -0.134 (-0.065) | 0.027 | 0.011 | -0.160 (-0.973) | 0.271 | <0.001 |
| **Frontal Cortical thickness** |  |  |  | 0.251 (3.250) | 0.695 | <0.001 |
|  | **Temporal cortical thickness** | | | **Language** | | |
| **Multiple lobar CMBs** | -0.069 (-0.058) | 0.024 | 0.016 | -0.056 (-1.383) | 1.171 | 0.017 |
| **Temporal cortical thickness** |  |  |  | 0.442 (22.166) | 2.795 | <0.001 |
|  | **Parietal cortical thickness** | | | **Visuospatial function** | | |
| **Multiple lobar CMBs** | -0.148 (-0.067) | 0.021 | 0.002 | -0.114 (-2.400) | 0.954 | 0.012 |
| **Parietal cortical thickness** |  |  |  | 0.180 (10.465) | 3.056 | <0.001 |
|  | **Temporal cortical thickness** | | | **Memory** | | |
| **Multiple lobar CMBs** | -0.069 (-0.059) | 0.024 | 0.014 | -0.120 (-2.848) | 2,158 | 0.002 |
| **Temporal cortical thickness** |  |  |  | 0.442 (44.662) | 5.128 | <0.001 |
|  | **Frontal cortical thickness** | | | **Frontal executive function** | | |
| **Multiple lobar CMBs** | -0.160 (-0.055) | 0.139 | <0.001 | -0.160 (-14.300) | 4.861 | 0.004 |
| **CSS** | -0.134(-0.067) | 0.027 | 0.012 | -0.135(-15.094) | 5.846 | <0.001 |
| **Frontal Cortical thickness** |  |  |  | 0.224 (44.062) | 15.298 | 0.004 |
|  | **Total cortical thickness** | | | **MMSE** | | |
| **Multiple lobar CMBs** | - 0.199 (-0.489) | 0.020 | <0.001 | -0.042 (-1.329) | 0.568. | <0.001 |
| **CSS** | -0.144 (-0.064) | 0.026 | 0.013 | -0.110 (-2.228) | 0.738 | 0.016 |
| **Total Cortical thickness** |  |  |  | 0.390 (15.550) | 1.647 | <0.001 |

**CMBs** cerebral microbleeds; **CSS** cortical superficial siderosis; **MMSE** mini mental state examination; **β** beta coefficiency; **SE** standard error; ***P*** *P-*value
